# Modelling the geographical distribution of soil-transmitted helminth infections in Bolivia

**DOI:** 10.1186/1756-3305-6-152

**Published:** 2013-05-25

**Authors:** Frédérique Chammartin, Ronaldo GC Scholte, John B Malone, Mara E Bavia, Prixia Nieto, Jürg Utzinger, Penelope Vounatsou

**Affiliations:** 1 Department of Epidemiology and Public Health, Swiss Tropical and Public Health Institute, P.O. Box, , CH-4002 Basel, Switzerland; 2 University of Basel, P.O. Box, , CH-4003 Basel, Switzerland; 3 School of Veterinary Medicine, Louisiana State University, Baton Rouge, LA, 70803, USA; 4 Preventive Medicine Department, Federal University of Bahia, Salvador, Bahia, 40110-060, Brazil

**Keywords:** Bayesian modelling, Bolivia, Geostatistical variable selection, Mapping, Soil-transmitted helminths

## Abstract

**Background:**

The prevalence of infection with the three common soil-transmitted helminths (i.e. *Ascaris lumbricoides*, *Trichuris trichiura*, and hookworm) in Bolivia is among the highest in Latin America. However, the spatial distribution and burden of soil-transmitted helminthiasis are poorly documented.

**Methods:**

We analysed historical survey data using Bayesian geostatistical models to identify determinants of the distribution of soil-transmitted helminth infections, predict the geographical distribution of infection risk, and assess treatment needs and costs in the frame of preventive chemotherapy. Rigorous geostatistical variable selection identified the most important predictors of *A. lumbricoides*, *T. trichiura*, and hookworm transmission.

**Results:**

Results show that precipitation during the wettest quarter above 400 mm favours the distribution of *A. lumbricoides*. Altitude has a negative effect on *T. trichiura*. Hookworm is sensitive to temperature during the coldest month. We estimate that 38.0%, 19.3%, and 11.4% of the Bolivian population is infected with *A. lumbricoides*, *T. trichiura*, and hookworm, respectively. Assuming independence of the three infections, 48.4% of the population is infected with any soil-transmitted helminth. Empirical-based estimates, according to treatment recommendations by the World Health Organization, suggest a total of 2.9 million annualised treatments for the control of soil-transmitted helminthiasis in Bolivia.

**Conclusions:**

We provide estimates of soil-transmitted helminth infections in Bolivia based on high-resolution spatial prediction and an innovative variable selection approach. However, the scarcity of the data suggests that a national survey is required for more accurate mapping that will govern spatial targeting of soil-transmitted helminthiasis control.

## Background

Soil-transmitted helminth infections are mainly caused by the intestinal worms *Ascaris lumbricoides*, *Trichuris trichiura*, and the two hookworm species *Ancylostoma duodenale* and *Necator americanus*[1]. They are the most prevalent neglected tropical diseases, and they are widely distributed across Latin America [2,3]. Soil-transmitted helminthiasis and other neglected tropical diseases primarily affect low-income populations, causing chronic conditions, learning disabilities, and reduced productivity and income earning capacity in later life. Morbidity control and, where resources allow, local elimination are now recognised as a priority for achieving the millennium development goals [4]. In 2009, the Pan American Health Organization (PAHO) developed a plan to eliminate neglected and other poverty-related diseases in Latin America and Caribbean countries. Soil-transmitted helminthiases were identified as target diseases to be controlled through preventive chemotherapy and by promoting access to clean water, improved sanitation, and better hygiene behaviour [5]. Control programmes require reliable baseline information of the geographical distribution of the number of infected people and disease burden estimates in order to enhance the spatial targeting and cost-effectiveness of planned interventions [6,7].

Bolivia is ranked last among the Western Hemisphere countries in terms of key health indicators. For example, child mortality rate is the worse in South America and, according to the 2001 census, 64% of the population did not have enough income to meet their basic needs [8]. The prevalence of soil-transmitted helminth infection is estimated at around 35% [9]. However, the geographical distribution and burden of soil-transmitted helminth infections is poorly documented.

In the past 20 years, progress in geographical information system (GIS) and remote sensing techniques, coupled with spatial modelling, enabled a better understanding of helminth ecology and mapping at high spatial resolution [6,7,10-13]. Ecological niche and biology-driven models have been used in assessing the distribution of helminth infections [14-16]. Bayesian geostatistical models offer a robust methodology for identifying determinants of the disease distribution and for predicting infection risk and burden at high spatial scales [17]. These models have been widely used in assessing the relationship between helminth infection with demographic, environmental, and socioeconomic predictors, at sub-national [11,18], national [19], or regional scales [13,20,21]. In the Americas, high resolution, geostatistical, model-based risk estimates have been obtained for the whole continent [22] as well as for Brazil [23]. A key issue in geostatistical modelling is the selection of the predictors. Most of the variable selection methods in geostatistical applications rely on standard methods, such as stepwise regression or bivariate associations that are appropriate for non-spatial data [10,11]. However, ignoring spatial correlation leads to incorrect estimates of the statistical significance of the predictors included in the model. Recently, Bayesian variable selection has been introduced in geostatistical disease mapping [21,24].

The purpose of this paper was to map the geographical distribution of *A. lumbricoides*, *T. trichiura*, and hookworm in Bolivia, and to estimate the risk, number of infected school-aged children, and the costs related to treatment interventions in the country. Survey data were extracted from published and unpublished sources. Bayesian geostatistical models were employed using rigorous variable selection procedures.

## Methods

### Disease data

Data on the prevalence of soil-transmitted helminth infection were extracted from the global neglected tropical diseases (GNTD) database (http://www.gntd.org) [13,16,21,22,25]. The GNTD database is an open-access platform consisting of geo-referenced survey data pertaining to schistosomiasis, soil-transmitted helminthiasis, and other neglected tropical diseases. Surveys are identified through systematic searches of electronic databases such as PubMed and ISI Web of Knowledge with no restriction of publication date or language. Our search strategy, including data quality appraisal, is summarised in Table 1.

**Table 1:** Search strategy identification of for soil-transmitted helminth infection prevalence survey data in Bolivia

**Keywords**	**Exclusion criteria**	**Quality control measures**
Bolivi* AND helminth* (OR ascari*, OR trichur*, OR hookworm, OR necator, OR ankylostom*,OR ancylostom*,OR strongy*, OR hymenolepis*, OR toxocara*, OR enterobius*, OR geohelminth*, OR nematode)	Hospital-based study; case-control study (except control group); clinical trials (except baseline); drug-efficacy study (except placebo group); displaced population (travellers, military, expatriates, nomads); population treated for the infection during the past year; unclear location of the survey; sample size <10	Double check of each entry; search and elimination of duplicates; recalculation of prevalence; verification in Google Maps that point level coordinates correspond to human settlement

### Environmental, socioeconomic, and population data

A total of 40 environmental and socioeconomic variables were considered in our analysis. Environmental variables included 19 interpolated climatic data from weather stations related to temperature and precipitation, vegetation proxies such as the enhanced vegetation index (EVI) and normalized difference vegetation index (NDVI), altitude, land cover, as well as information on soil acidity and soil moisture. Various unsatisfactory basic needs (UBN) poverty indicators related to adequate housing material, insufficient housing space, inadequate services of water and sewer systems and inadequate health attention were used as proxies of poverty. In addition, human development index (HDI) and infant mortality rate (IMR) were considered as alternative poverty measures. Impact of direct human influence on ecosystems was accounted by human influence index (HII). Population density and the proportion of school-aged children (age: 5–14 years), were used to estimate treatment needs and costs of intervention. Sources of the variables, together with their spatial and temporal resolution, are summarised in Table 2.

**Table 2:** Data sources and properties of the predictors explored to model soil-transmitted helminth infection risk in Bolivia

**Data type**	**Source**	**Date**	**Temporal resolution**	**Spatial resolution**
19 climatic variables related to temperature and precipitation	WorldClim^1^	1950-2000	-	1 km
Altitude	SRTM^2^	2000	-	1 km
Land cover	MODIS/Terra^3^	2000-2011	Yearly	1 km
EVI / NDVI	MODIS/Terra^3^	2000-2011	16 days	1 km
Soil acidity / soil moisture	ISRIC-WISE^4^	1960-2000	-	10 km
Unsatisfactory basic needs (UBN)	Census^5^	2001	10 years	Municipality
Infant mortality rate (IMR)	CIESIN^6^	2005	Yearly	5 km
Human influence index (HII)	LTW^7^	2005	-	1 km
Human development index (HDI)	PAHO^8^	2005	-	Municipality
Population density	WISE3^4^	2010	-	10 km
School-aged children proportion	IDB^9^	2010	-	Country

^1^ WorldClim Global Climate database v.1.4; available at: http://www.worldclim.org/ (accessed: 1 March 2012).

^2^ Shuttle Radar Topography Mission (SRTM); available at: http://www.worldclim.org/ (accessed: 1 March 2012).

^3^ Moderate Resolution Imaging Spectroradiometer (MODIS); available at: https://lpdaac.usgs.gov/ (accessed: 15 December 2012).

^4^ Global soil profile data ISRIC-WISE database v.1.2; available at: http://www.isric.org/ (accessed: 15 December 2012).

^5^ Instituto nacional de estadística, 2001 census; available at: http://www.ine.gob.bo/ (accessed: 1 March 2012).

^6^ 2005 Global subnational infant mortality rates, Center for International Earth Science Information Network (CIESIN). CIESIN, Palisades, NY, USA; available at: http://www.ciesin.columbia.edu/povmap/ds_global.html (accessed: 1 March 2012).

^7^ Last of the Wild Data Version 2, 2005 (LTW-2): Global Human Footprint Dataset (Geographic). Wildlife Conservation (WCS) and Center for International Earth Science Information Network (CIESIN); available at: http://www.ciesin.org/wildareas/ (accessed: 1 March 2012).

^8^ Pan American Health Organization; personal communication.

^9^ International Data Base (IDB) United States Census Bureau; available at: http://www.census.gov/population/international/ (accessed: 1 March 2012).

For prediction purposes, a 5 × 5 km spatial resolution grid was created. Environmental data available at 1 × 1 km spatial resolution, were averaged over their closest neighbours. Soil acidity, soil moisture, and infant mortality rate were linked to the prediction pixel with the closest distance. UBN and HDI were re-scaled by assigning to each grid pixel the value of the administrative unit they belong to. Re-scaling was performed in ArcMap version 10.0 (Environmental Systems Research Institute; Redlands, CA, USA).

### Geostatistical model

Disease survey data are typically binomially distributed and modelled via a logistic regression. More precisely, let Y_*i*_, *n*_*i*_, and *p*_i_ be the number of infected individuals, the number of individuals screened, and the prevalence or risk of infection at location *i*, respectively, such as *Y*_*i*_ ~ *Bn *(* n*_*i*,_* p*_*i*_). Spatial correlation is taken into account by introducing location-specific parameters *φ*_*i*_ that are considered as unobserved latent data from a stationary spatial Gaussian process. We modelled a temporal trend, the selected predictors (i.e. environmental and socioeconomic factors) X_*i*_ and φ_*i*_ on the *logit* scale: *logit*(*p*_*i*_) = *X*_*i*_^*T*^*β* + *φ*_*i*_. The temporal trend was modelled by a binary variable T_*i*_ indicating whether a survey was carried out before or from 1995 onwards. We assumed that $\underline{\varphi }\sim \mathrm{MVN}\;\left(\underline{0},\Sigma \right)$ with variance-covariance matrix *Σ*. Geographical correlation was modelled by an isotropic exponential correlation function of distance, i.e. $\Sigma_{\mathrm{cd}}=\sigma_{\mathrm{sp}}^{2}\text{exp}\left(-\rho d_{\mathrm{cd}}\right)$, where *d*_*cd*_ is the Euclidean distance between locations *c* and *d*, *σ*_*sp*_^2^ is the geographical variability known as the partial sill, and *ρ* is a smoothing parameter controlling the rate of correlation decay. The geographic dependency (range) was defined as the minimum distance at which spatial correlation between locations is less than 5% and is calculated by 3/*ρ*. To facilitate model fit, the model was formulated using a Bayesian framework of inference. Vague normal prior distributions $\underline{\beta }\sim N\left(0,\sigma^{2}I\right)$ were adopted for the regression coefficients, an inverse gamma distribution $\sigma_{\mathrm{sp}}^{2}\sim \mathrm{IG}\left(a_{\sigma_{\mathrm{sp}}^{2}},b_{\sigma_{\mathrm{sp}}^{2}}\right)$ was chosen for the variance *σ*_*sp*_^2^, and a gamma distribution was assumed for the spatial decay *ρ, ρ* ~ *G*(*a*_*ρ*_, *b*_*ρ*_).

### Geostatistical variable selection

Bayesian stochastic search variable selection [26] was performed to select the most important predictors among the 40 socioeconomic and environmental predictors, while taking into account the spatial correlation in the data. Predictors were either standardised or categorised if they presented a non-linear bivariate association with the observed helminthiasis prevalence (on the *logit* scale). Furthermore, we considered a spike and slab prior distribution for the regression coefficients [27], which improves convergence properties of the Markov chain Monte Carlo (MCMC) simulation and allows selection of blocks of covariates such as categorical ones. In addition, we assessed correlation between the predictors and forced the model to choose only one (or none) predictor among those highly correlated (i.e. absolute value of Pearsons correlation coefficient larger than 0.9). The geostatistical variable selection explores all possible models and the final model is the one presenting the highest posterior probability.

**Figure 1:**
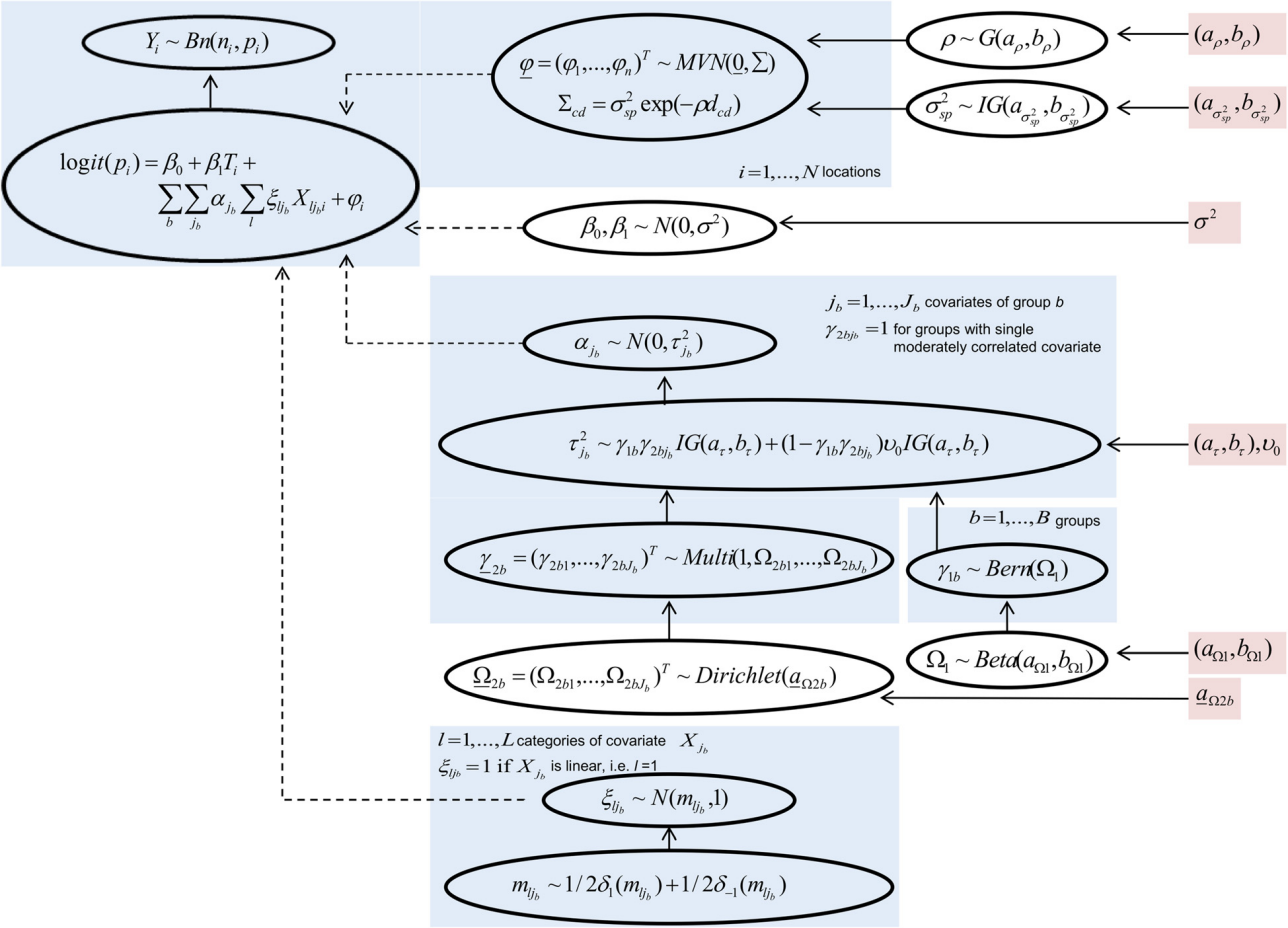
**Acyclic graph of the geostatistical variable selection.** Stochastic and logical nodes are represented as ellipses. Dashed arrows are logical links and straight line arrows are stochastic dependencies. Fixed parameters of the prior distributions are highlighted in pink.

The geostatistical variable selection specification is summarised in Figure 1. In particular, predictors were classified into 19 groups *b*, (*b* = 1, …, 19), depending on their mutual correlations. Thirteen predictors that were only moderately correlated with any other predictors were separated into single variable groups. Highly correlated predictors were divided into six groups, each containing 38 variables $X_{j_{b}},j_{b}=1,\ldots ,J_{b}$. The regression coefficients are defined as the product of an overall contribution $\alpha_{j_{b}}$ of the predictor $X_{j_{b}}$ and the effect $\xi_{lj_{b}}$ of each of its elements (i.e. categories), $X_{lj_{b}},l=1,\ldots ,L$ categories (excluding baseline) of the predictor $X_{j_{b}}$. We assigned a spike and slab prior [27,28], which is a scaled normal mixture of inverse-gamma to $\alpha_{j_{b}}$, that is $\alpha_{j_{b}}\sim N\left(0,\tau_{j_{b}}^{2}\right)$, where $\tau_{j_{b}}^{2}∼\gamma_{1b}\;\gamma_{2j_{b}}\;\mathrm{IG}\left(a_{\tau },b_{\tau }\right)+\left(1-\gamma_{1b}\;\gamma_{2j_{b}}\right)\;\upsilon_{0}\mathrm{IG}\left(a_{\tau },b_{\tau }\right)$.ɑ_τ_ and *b*_τ_ are fixed parameters of non-informative inverse-gamma distribution, while *υ*_*0*_ is a small constant shrinking $\alpha_{j_{b}}$ to zero when the predictor is excluded. The presence or absence of the predictors is defined by the product of two indicators *γ*_1*b*_ and ${\underline{\gamma }}_{2b}={\left(\gamma_{2b1},\ldots ,\gamma_{2bJ_{b}}\right)}^{T}$, where *γ*_1*b*_ determines the presence or absence of the group *b* in the model and ${\underline{\gamma }}_{2b_{j}},j_{b}=1,\ldots ,j_{b}$ allows selection of a single predictor within the group. A Bernoulli and a multinomial prior distribution are assigned to *y*_1*b*_ and *γ*_2*b*_, respectively, such as *γ*_1*b*_ ~ *Bern*(*Ω*_1_) and ${\underline{\gamma }}_{2b}\sim \mathrm{Multi}\left(1,\Omega_{2b1},\ldots ,\Omega_{2bJ_{b}}\right)$ with inclusion probabilities *Ω*_1_ and ${\underline{Ω}}_{2b}$. To allow greater flexibility in estimating model size, these probabilities are considered as hyper-parameters having non-informative beta and Dirichlet distributions. A mixture of two Gaussian distributions is assumed for $\xi_{l_{j}{}_{b}},\xi_{l_{j}{}_{b}}\sim N\left(m_{{}_{l_{j}{}_{b}}},1\right),m_{{}_{l_{j}{}_{b}}}\sim 1/2\delta_{1}\left(m_{{}_{l_{j}{}_{b}}}\right)+1/2\delta_{-1}\left(m_{{}_{l_{j}{}_{b}}}\right)$, which shrinks $\xi_{l_{j}{}_{b}}$ towards |1| (multiplicative identity). For predictors moderately correlated, $\gamma_{2bj_{b}}$ is fixed to 1, while the effect of linear predictors is only defined by an overall contribution of α.

To complete model specification, the spatial random effect *φ* is modelled as defined in the previous subsection and a vague normal distribution is assigned to the constant term of the model. The subset of variables included in the models with the highest posterior probabilities identified the final models.

### Implementation details

We considered the following values for the parameters of the prior distributions: σ^2^=100, (ɑ_*ρ,*_*b*_*ρ*_)*=*(0*.*01,0.01), $\left(a_{\sigma_{\mathrm{sp}}^{2}},b_{\sigma_{\mathrm{sp}}^{2}}\right)=\left(2.01,\;1.01\right)$,(ɑ_*τ*_,*b*_*τ*_)=(5,25), (ɑ_Ω1_, *b*_Ω1_)=(1,1), $\left({\underline{a}}_{\Omega 2b}\right)=\left(1,\ldots ,1\right)$ and υ_0_=0.00025.

MCMC simulations were used to estimate model parameters. For variable selection, a burn-in of 50,000 iterations was performed and another 50,000 iterations were run to identify the model with the highest posterior probability. For each infection, the best geostatistical model was fitted with one chain sampler and a burn-in of 5,000 iterations. Convergence was assessed after an average of 50,000 iterations using the Raftery and Lewis [29] diagnostics. A posterior sample of 1,000 values was used for validation purposes and for prediction at un-sampled locations. Prediction was carried out using Bayesian kriging [17] over a grid of 26,519 pixels of 5 × 5 km spatial resolution. The median and standard deviation of the predicted posterior distribution were plotted to produce smooth risk maps together with their uncertainty. Analyses were implemented in WinBUGS 14 (Imperial College and Medical Research Council; London, UK), while R version 2.7.2 (The R Foundation for Statistical Computing) was used for predictions. Non-spatial explorative statistical analyses were performed in Stata version 10.0 (Stata Corporation; College Station, USA).

### Model validation

Models were fitted on a random training sample of 39 locations for *A. lumbricoides* and *T. trichiura*, and 37 locations for hookworm. Model validation was performed on the remaining 10 test locations (around 20% of the total locations). The predictive performance was calculated by the proportion of test locations being correctly predicted within the *k*^th^ Bayesian credible interval (BCI) of the posterior predictive distribution (limited by the lower and upper quantiles $\mathrm{BC}I_{i\left(k\right)}^{l}$ and $\mathrm{BC}I_{i\left(k\right)}^{u}$, respectively), where *k* indicates the probability coverage of the interval as: $\frac{1}{10}\sum_{i=1}^{10}\min\left(I\left(\mathrm{BC}I_{i\left(k\right)}^{l}<p_{i}\right),I\left(\mathrm{BC}I_{i\left(k\right)}^{u}>p_{i}\right)\right)$ The higher the number of test locations within the narrowest and smallest coverage BCI, the better the model predictive ability.

### Treatment needs and estimated costs

The number of infected school-aged children was calculated for each pixel from the geostatistical model-based estimated risk and the population density. According to guidelines put forward by the World Health Organization (WHO), all school-aged children should be treated twice a year in high-risk communities (prevalence of any soil-transmitted helminth infection ≥50%) and once every year in low-risk communities (prevalence of any soil-transmitted helminth infection between 20% and 50%). Large-scale preventive chemotherapy is not recommended in areas where prevalence is less than 20%; indeed treatment should be delivered on a case-by-case basis in such areas [30]. We estimated the number of albendazole or mebendazole treatments needed during one year in the school-aged population, considering different units at which levels of risk were determined (i.e. pixel, municipality, province, and department). Hence, we followed the same methodology as for estimating annualised praziquantel needs against schistosomiasis [31]. To calculate the cost of a school-based deworming programme in Bolivia, the estimated number of treatments was multiplied by an average unit cost equivalent to US$ 0.25, which includes additional expenses for training, drug distribution, and administration [9,32].

## Results

Seven out of 59 identified peer-reviewed publications reported soil-transmitted helminth infection prevalence data in Bolivia [33-39]. For the current investigation, additional data were obtained from a 2006 report of the Ministry of Health (MoH) in Bolivia [40].

**Figure 2:**
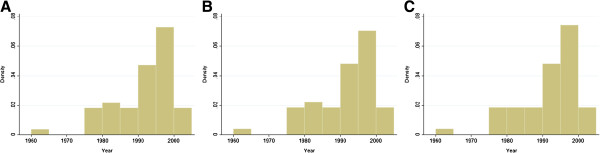
**Frequency distribution of the survey periods in Bolivia for *A. lumbricoides *(A), *T. trichiura *(B), and hookworm (C).**

We obtained relevant prevalence data for *A. lumbricoides*, *T. trichiura,* and hookworm for 49, 49, and 47 survey locations, respectively, covering the period from 1960 to 2010. The frequency distribution of the surveys, stratified by helminth species, is given in Figure 2. Six surveys out of 49 were reported at municipality level (administrative level 3) and were assigned to the centroid of their municipality. The remaining 43 locations were reported at school or village level and were therefore considered as point data. Most of the studies (71%) explicitly screened school-aged children (the remaining studies are either referring to entire populations or provide no information on the age range of the participants). With regard to the diagnosis of soil-transmitted helminthiasis, 47% of the studies used the WHO-recommended Kato-Katz technique [41], whereas in 21 locations the diagnostic approach was not stated, and in five locations other diagnostic techniques were utilised.

**Figure 3:**
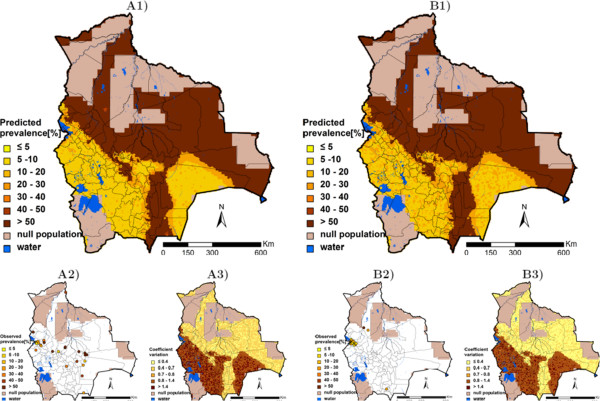
***Ascaris lumbricoides *infection risk in Bolivia.** The maps show the situation before 1995 (**A**) and from 1995 onwards (**B**), and provide estimates of the geographical distribution of the infection (1), the observed prevalence (2), and the coefficient of variation (3).

**Figure 4:**
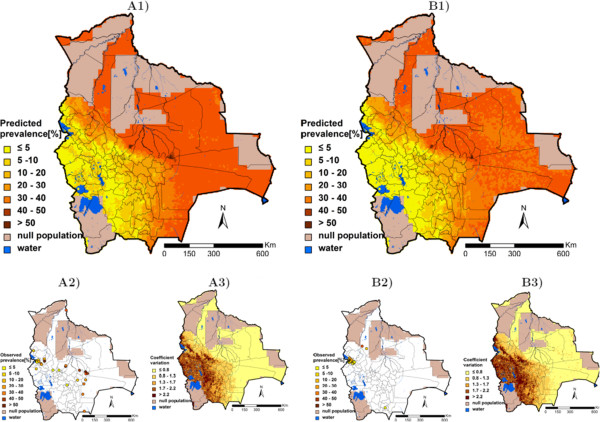
***Trichuris trichiura *infection risk in Bolivia.** The maps show the situation before 1995 (**A**) and from 1995 onwards (**B**), and provide estimates of the geographical distribution of the infection (1), the observed prevalence (2), and the coefficient of variation (3).

**Figure 5:**
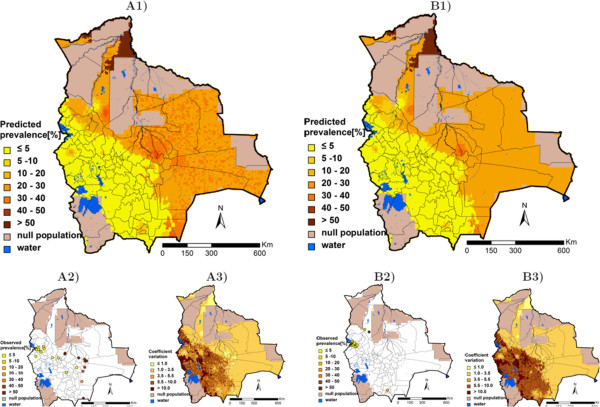
**Hookworm infection risk in Bolivia.** The maps show the situation before 1995 (**A**) and from 1995 onwards (**B**), and provide estimates of the geographical distribution of the infection (1), the observed prevalence (2), and the coefficient of variation (3).

**Figure 6:**
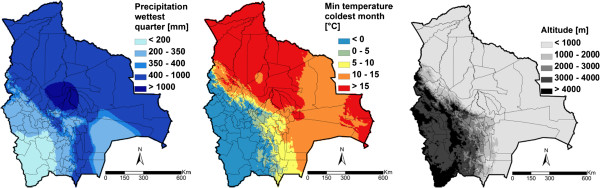
Major climatic zones and spatial distribution of the remotely sensed predictors in Bolivia.

**Figure 7:**
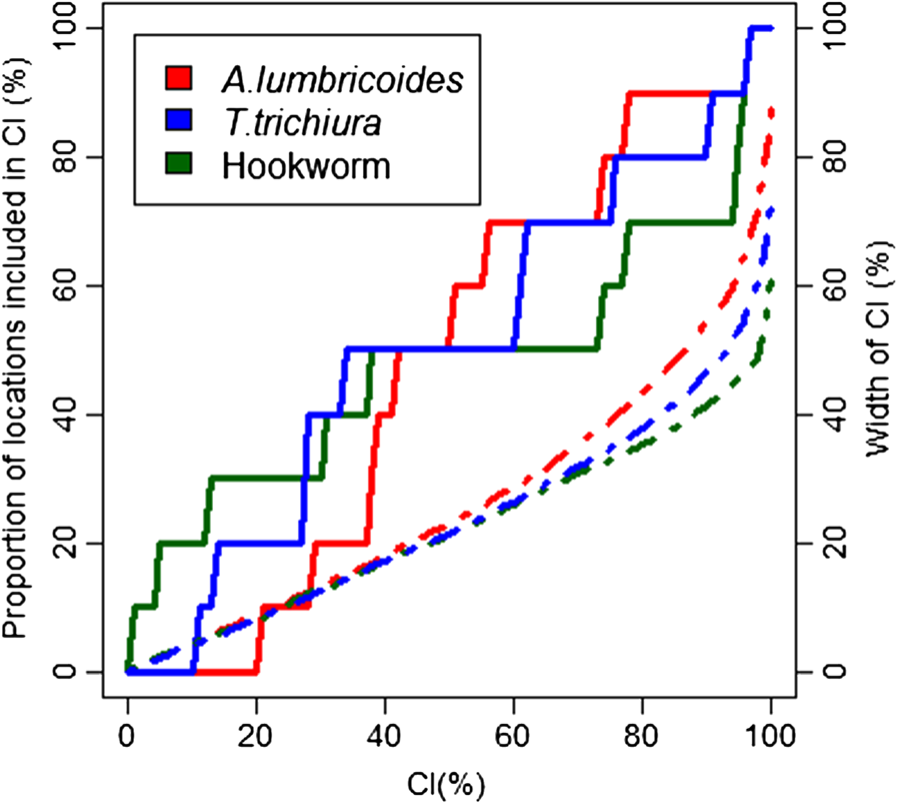
Proportion of locations with observed prevalence falling within credible intervals of the posterior predictive distribution with probability coverage varying from 1% to 100%.

Table 3 summarises, for each helminth species, the three best models resulting from the geostatistical variable selection. For *A. lumbricoides*, the model based on precipitation of the wettest quarter has the highest posterior probability of 42.2%. For *T. trichiura* the best model included altitude (posterior probability = 10.1%), while for hookworm, the model with the highest posterior probability (10.2%) included the minimum temperature during the coldest month. Results of the geostatistical logistic regressions, together with estimates of the bivariate non-spatial associations, are presented in Table 4. Precipitation of the wettest quarter above 400 mm had a positive effect on the odds of *A. lumbricoides* infection risk; hookworm infection risk was positively associated to the minimum temperature during the coldest month, and the higher the altitude, the lower the odds of *T. trichiura* infection. Although the risk of infection with the three helminth species decreased after 1995, this effect was not important in the spatial models as reflected by the 95% BCI of the odds ratio estimates. Figures 3, 4, and 5 show the geographical distribution of the predicted risks for each of the three soil-transmitted helminth species before and after 1995, the corresponding standard deviation of the predictive distribution and the raw survey data. Maps of all predictors involved in the final geostatistical models are shown in Figure 6. Bolivia presents generally a lower risk of soil-transmitted helminthiasis in the south-western part of the country, where high altitude brings unsuitable climatic conditions for the development of the parasites. For the three soil-transmitted helminth infections, the maps of the posterior standard deviation reflect the pattern of the predicted risk. However, we note that for hookworm, where the spatial correlation is more important (spatial range estimated to 128.4 km), the standard deviation was also low in areas surrounding the survey locations, suggesting less uncertainty in the estimation of the spatial random effect in the neighbourhood of observed data. Figure 7 shows that the risks of *A. lumbricoides*, * T. trichiura* and hookworm infection are correctly predicted within 95% BCIs for 90%, 90%, and 80%, respectively.

**Table 3:** Variables selected by the geostatistical variable selection approach

	***A. lumbricoides *infection**			***T. trichiura *infection**			**Hookworm infection**		
**Group 1**									
Home with indoor plumbing^1^	0	0	0	0	0	0	0	0	0
People with drinking water at home^1^	0	0	0	0	0	0	0	0	0
Pipe network	0	0	0	0	0	0	0	0	0
Population with high quality of life	0	0	0	0	0	0	0	0	0
Population with UBN	0	0	0	0	0	0	0	0	0
Population with sanitation at home	0	0	0	0	X	0	0	0	0
**Group 2**									
Population with material UBN	0	0	0	0	0	0	0	0	0
Population with low quality of life	0	0	0	0	0	0	0	0	0
**Group 3**									
Minimum temperature coldest month^1,2^	0	0	0	0	0	0	X	0	X
Altitude	0	0	0	X	0	0	0	0	0
Annual temperature	0	0	0	0	X	0	0	0	0
Maximum temperature warmest month	0	0	0	0	0	0	0	0	0
Temperature wettest quarter	0	0	0	0	0	0	0	X	0
Temperature driest quarter	0	0	0	0	0	0	0	0	0
Temperature warmest quarter	0	0	0	0	0	0	0	0	0
Temperature coldest quarter	0	0	0	0	0	0	0	0	0
**Group 4**									
Temperature annual range^3^	0	0	0	0	0	0	0	0	0
Temperature diurnal range	0	0	0	0	0	X	0	0	0
**Group 5**									
Annual precipitation^1,2,3^	0	0	0	0	0	0	0	0	0
Precipitation wettest month^1,2^	0	0	0	0	0	0	0	0	0
Precipitation wettest quarter^1,2^	X	0	0	0	0	0	0	0	0
Precipitation driest month^2,3^	0	0	0	0	0	0	0	0	0
Precipitation driest quarter^2^	0	0	0	0	0	0	0	0	0
Precipitation warmest quarter^3^	0	0	0	0	0	0	0	0	0
Precipitation coldest quarter^2^	0	0	0	0	0	0	0	0	0
**Group 6**									
Enhanced vegetation index	0	0	0	0	0	0	0	0	0
Normalized difference vegetation index	0	0	0	0	0	0	0	0	X
**Moderately correlated**									
Soil acidity^1,3^	0	0	X	0	0	0	0	0	0
Precipitation seasonality^1,3^	0	0	0	0	0	0	0	0	0
Soil moisture^2^	0	0	0	0	0	0	0	0	0
Isothermality	0	0	0	0	0	0	0	0	0
Temperature seasonality	0	0	0	0	0	0	0	0	0
Human influence index	0	0	0	0	0	0	0	0	0
Infant mortality rate	0	0	0	0	0	0	0	0	0
Human development index	0	0	0	0	0	0	0	0	0
Population with education UBN	0	0	0	0	0	0	0	0	0
Population with overcrowding UBN	0	0	0	0	0	0	0	0	0
Population with sanitation UBN	0	0	0	0	0	0	0	0	0
Population with light at home	0	0	0	0	X	0	0	0	0
Unemployment rate	0	0	0	0	0	0	0	0	0
**Posterior probability [%]**	42.2	5.9	2.9	10.1	6.0	5.2	10.2	4.7	2.0

^1^Categorised for *A. lumbricoides;*^2^categorised for *T. trichiura*; ^3^categorised for hookworm; X (selected), 0 (not selected).

The best three models selected by the geostatistical variable selections are presented for each soil-transmitted helminth species, together with their posterior probabilities.

**Table 4:** Parameter estimates of non-spatial bivariate and Bayesian geostatistical logistic models with environmental and socio-economic predictors

	**Bivariate non-spatial**		**Geostatistical model**	
	**OR**^**†**^	**95% CI**^**†**^	**OR**^**†**^	**95% BCI**^**†**^
***A. lumbricoides *infection**				
Survey period				
Before 1995	1.00		1.00	
1995 onwards	0.26	(0.24; 0.29)^*^	0.94	(0.64; 1.42)
Precipitation wettest quarter (mm)				
<350	1.00		1.00	
350-400	1.42	(1.23; 1.66)^*^	1.32	(0.56; 2.81)
≥400	12.25	(10.95; 13.70)^*^	12.52	(5.05; 25.56)^*^
			**Median**	**95% BCI**^**†**^
**σ **^2^_sp_			1.11	(0.72; 2.00)
Range (km)			9.2	(1.3; 63.0)
***T. trichiura *infection**				
Survey period				
Before 1995	1.00		1.00	
1995 onwards	0.33	(0.29; 0.37)^*^	0.85	(0.55; 1.30)
Altitude	0.33	(0.31; 0.36)^*^	0.37	(0.26; 0.56)^*^
			**Median**	**95% BCI**^**†**^
**σ **^2^_sp_			1.29	(0.77; 2.23)
Range (km)			28.7	(3.2; 80.2)
**Hookworm infection**				
Survey period				
Before 1995	1.00		1.00	
1995 onwards	0.45	(0.41; 0.50) ^*^	0.72	(0.12; 4.19)
Minimum temperature coldest month	6.25	(5.81; 6.72)^*^	11.35	(5.00; 22.20) ^*^
			**Median**	**95% BCI**^**†**^
**σ **^2^_sp_			3.07	(1.50; 7.44)
Range (km)			128.4	(39.8; 387.5)

^**†**^OR: odds ratio; 95% CI: lower and upper bound of a 95% confidence interval; 95% BCI: lower and upper bound of a 95% Bayesian credible interval.

^*^Significant based on 95% CI or 95% BCI.

Table 5 shows the total amount of treatment required on a yearly basis and the associated cost when the calculation is based on soil-transmitted helminth infection risk estimates, aggregated to various administrative levels. The estimated number of children targeted increases from 1,481,605 to 2,180,101, depending on the administrative level at which the risk is aggregated. However, the number of treatments required remains quite stable, indicating large spatial heterogeneity of the infection risk within the units. Model-based predictions and estimates of number of school-aged children infected with the three soil-transmitted helminth species, aggregated at province and country level, are presented in the Additional file 1. The estimated prevalence for *A. lumbricoides*, *T. trichiura*, and hookworm infection is 38.0%, 19.3%, and 11.4%, respectively. Taking the three soil-transmitted helminth species together, we estimate that 48.4% of the school-aged population is infected with at least one species, assuming independence of the three soil-transmitted helminth infections. The highest number of school-aged children needing treatment is concentrated in the densely populated Andrés Ibáñez province, while the highest risk for the three soil-transmitted helminths taken together is predicted for the Vaca Díez province.

**Table 5:** Yearly estimation of school-aged children needing preventive chemotherapy against soil-transmitted helminthiasis in Bolivia

	**5 × 5 km**	**Municipality**	**Province**	**Department**
Number of children targeted	1,481,605	1,749,136	1,907,658	2,180,101
Number of treatment required	2,894,936	2,868,016	2,847,604	3,013,413
Cost (US$)	723,734	717,003	711,901	753,353

Estimates are based on prevalence predicted at pixels of 5 × 5 km resolution and aggregated over different administrative levels.

## Discussion

We present spatially explicit estimates of the risk and number of school-aged children infected with the three common soil-transmitted helminths in Bolivia using a rigorous geostatistical variable selection approach. Survey data were extracted from the literature, geo-referenced, and made public via the open-access GNTD database. Our study also identified important data needs and gaps. For example, most of the surveys were conducted along the sub-Andean region. On the other hand, only few survey locations were available in the less densely populated highlands and in the northern tropical areas. Rigorous geostatistical variable selection methods have been used to identify environmental and socioeconomic determinants that govern the distribution of soil-transmitted helminth infection in Bolivia. The country, nestled between the high Andean peaks (on the West) and the Amazon forest (on the East), presents specific ecological characteristics that shape helminth cycles in a complex way. High altitude and diverse topography, as well as the paucity of weather stations in remote areas can introduce interpolation bias in the climatic factors used in our analysis [42]. Bayesian variable selection helped in identifying the potential factors influencing the geographical distribution of the three common soil-transmitted helminth species. Our methodology enabled us to explore all possible models arising from 40 climatic and socioeconomic predictors, while accounting for spatial correlation in the data.

The parameterisation of the prior distribution of the regression coefficients as developed in this manuscript selects the best predictors among highly correlated ones, while addressing non-linearity. The selected predictors are plausible in terms of helminth biology, ecology, and epidemiology. Indeed, the distribution of *A. lumbricoides* was positively associated with precipitation above 400 mm during the wettest month. High humidity is related with faster development of parasite eggs in the free environment. Low humidity, on the other hand, can cease embryonation of *A. lumbricoides*[43,44]. The positive association between the minimum temperature of the coldest month and the prevalence of hookworm reflects inhibition of the development of the eggs by hostile cold temperatures [3,45]. The preventive effect of high altitude on *T. trichiura* infection risk has already been highlighted and explained by subsequent unfavourable temperature, which limits the transmission [46]. The three soil-transmitted helminth infection risks did not decrease significantly over time and we are unsure whether Bolivia has implemented integrated control measures. In the absence of preventive chemotherapy and/or sanitation improvement, environmental contamination is considerable, which may explain our observations of fairly constant infection rates over time [47,48].

The transmission of soil-transmitted helminthiasis occurs via contaminated food or fingers (*A. lumbricoides* and *T. trichiura*), or through the skin by walking on larvae-infested soil (hookworm). People living in poor conditions are more exposed due to their living conditions, the lack of access to clean water, sanitation, and health facilities [49]. Hence, we would have expected soil-transmitted helminth infections to be associated with some of the socioeconomic factors investigated, such as the ones related to sanitation [50]. However, none of the socioeconomic variables were picked up by our geostatistical variable selection approach. This may indicate that our socioeconomic proxies were not able to capture the socioeconomic disparities across the country when aggregated at district or municipality scales. Historical data are aggregated over villages or larger areas and they are rarely available at household level. Often variation in socioeconomic status is larger within rather than between locations, and hence, it may be harder for socioeconomic data to explain geographical differences.

Bolivian soil also exhibits specific characteristics such as presence of salt and soil compactation arising from livestock farming, which may affect the transmission of soil-transmitted helminths. In our analysis, we explored different soil predictors, including land cover, the vegetation indices EVI and NDVI, soil acidity and soil moisture. However, these factors failed to explain the distribution of the infection risks.

The population of Bolivia is mainly concentrated in and around the three main cities La Paz, Santa Cruz, and Cochabamba, where large parts of the country are uninhabited. The absence of human hosts breaks parasite life cycles. Thus, although environmental conditions may be suitable for parasite survival, there is no risk of transmission. To avoid potential misinterpretation, we clearly delineate areas where no humans live.

The predicted risk maps for the three common soil-transmitted helminth species in Bolivia should be interpreted with caution, particularly for areas characterised by only sparse survey data or poor coverage. Sample design is not optimised regarding the surveyed population; 29% of the data did not report the survey type (school-aged, community-based) and might bias the raw prevalence, as it is widely acknowledged that school-aged children are at higher risk of soil-transmitted helminths, particularly *A. lumbricoides* and *T. trichiura*, than their older counterparts [51]. Slightly less than half of the surveys stated the use of the WHO-recommended Kato-Katz technique for soil-transmitted helminth diagnosis [41,52]. Heterogeneity in the data regarding the sensitivities and specificities of the diagnostic methods might introduce measurement errors in the raw prevalence data. Furthermore, a zero hookworm prevalence was reported for 60% of the survey data. While these data suggest the non-endemicity of hookworm, the diagnostic approach might have underestimated the “true” prevalence due to diagnostic dilemmas [53,54]. Indeed, single Kato-Katz thick smears, low intensity infections, and delays in stool processing compromise sensitivity, particularly for hookworm diagnosis [55,56]. Giardina *et al.*[24] developed a zero-inflated binomial geostatistical model to estimate malaria burden when data contain a high proportion of zeros. This model could be adopted for soil-transmitted helminth infection and implemented in Bolivia as soon as more survey data become available. In addition, data in the literature usually report on hookworm prevalence, without differentiation of the species (*A. duodenale* and *N. americanus*). It would be interesting to analyse the two species separately, as they may have different ecological preferences.

Our study indicates that in Bolivia almost half (48.4%) of the population is infected with at least one of the three common soil-transmitted helminths. Our empirical-based estimates suggested that a total of 2,868,016 annualised treatments are required for preventive chemotherapy targeting school-aged children at the level of the municipalities. This estimate is higher than the one previously reported in the country (4,774,672 treatments for a 5-year campaign [9,32]). Population dynamic models [57-59] could be used to predict the effect of preventive chemotherapy on the epidemiological pattern of the three common soil-transmitted helminths, to evaluate the community effectiveness of the programme and to plan the duration of control interventions.

## Conclusions

In the framework of a preventive chemotherapy strategy, reliable maps of the distribution of infection risk and disease burden are needed to enhance cost-effectiveness of the interventions. Our high resolution estimates are based on existing data and their scarcity may raise doubts on the value of modelling of the disease distribution. However, soil-transmitted helminth infections are driven by environmental factors and, in the absence of interventions, the existing data can establish the relation between the risk of infection and climate. Hence, the risk maps produced are able to identify areas of high infection. Validation indicated that the models had good predictive ability. We therefore believe that the estimated maps can provide important inputs in the sampling design of a national survey by indicating the areas requiring more surveys. Hence, a coherent and optimally designed national survey is warranted to more accurately estimate the distribution and the number of people at risk of infection, so that preventive chemotherapy and other control measures can be optimally targeted.

## Abbreviations

BCI: Bayesian credible interval; CI: Confidence interval; EVI: Enhanced vegetation index; GIS: Geographical information system; GNTD: Global neglected tropical diseases (database); HDI: Human development index; HII: Human influence index; IMR: Infant mortality rate; MCMC: Markov chain Monte Carlo; MoH: Ministry of Health; NDVI: Normalized difference vegetation index; OR: Odds ratio; PAHO: Pan American Health Organization; UBN: Unsatisfactory basic needs; WHO: World Health Organization.

## Competing interests

The authors declare that they have no competing interests.

## Authors’ contributions

FC participated in data acquisition, analysed the data and wrote the manuscript. RGCS, JBM, MEB and PN participated in the environmental and socioeconomic data collection and helped interpreting their meaning. PV contributed to data analysis. PV and JU designed the study, helped interpreting the results, revised the manuscript and provided important intellectual content. All authors read and approved the manuscript.

## Supplementary Material

Additional file 1 Population-adjusted prevalence and estimated number of infected children (5–14 years old) with the three common soil-transmitted helminth (STH) infections, stratified by province and by country, for the period 1995 onwards, based on 2010 population estimates with 95% Bayesian credible interval (BCI). Click here for file
