# Supplementary material for: Modelling the geographical distribution of soil-transmitted helminth infections in Bolivia
Source: Parasit Vectors. 2013 May 25;6:152. doi: 10.1186/1756-3305-6-152 (PMC3681678; doi:10.1186/1756-3305-6-152)
Supplement: Additional file 1 — Population-adjusted prevalence and estimated number of infected children (5–14 years old) with the three common soil-transmitted helminth (STH) infections, stratified by province and by country, for the period 1995 onwards, based on 2010 population estimates with 95% Bayesian credible interval (BCI). [file 1756-3305-6-152-S1.pdf]

	Model-based prevalence (%)				Number of school-aged children infected*			
Province	*A. lumbricoides* infection	*T. trichiura* infection	Hookworm infection	STH infection**	*A. lumbricoides* infection	*T. trichiura* infection	Hookworm infection	STH infection**
Abel Iturralde	55.4 (45.6; 64.4)	34.5 (22.8; 48.1)	23.2 (7.3; 41.6)	69.9 (61.1; 78.6)	2,627 (2,164; 3,057)	1,635 (1,083; 2,283)	1,099 (347; 1,972)	3,316 (2,899; 3,728)
Alonso de Ibáñez	13.3 (8.8; 22.6)	6.8 (3.7; 12.4)	3.0 (0.5; 11.4)	20.9 (14.3; 29.4)	872 (574; 1,477)	443 (241; 811)	199 (35; 743)	1,363 (934; 1,925)
Andrés Ibáñez	55.8 (45.7; 63.8)	34.2 (24.2; 46.3)	20.7 (5.2; 46.6)	70.4 (61.6; 80.0)	180,358 (147,634; 206,402)	110,743 (78,296; 149,638)	66,809 (16,757; 150,726)	227,630 (199,126; 258,702)
Aniceto Arce	41.3 (34.8; 47.6)	22.9 (15.3; 31.4)	6.9 (1.4; 20.5)	52.5 (44.9; 59.5)	5,691 (4,796; 6,564)	3,149 (2,111; 4,321)	958 (193; 2,822)	7,229 (6,187; 8,194)
Antonio Quijarro	13.2 (8.9; 22.6)	6.0 (3.2; 11.3)	3.5 (0.7; 12.0)	20.3 (14.9; 28.8)	1,742 (1,180; 2,982)	798 (424; 1,487)	469 (98; 1,579)	2,686 (1,968; 3,806)
Arani	33.6 (26.7; 42.0)	16.5 (11.5; 23.7)	5.6 (1.3; 15.6)	42.2 (34.9; 49.5)	4,031 (3,201; 5,036)	1,984 (1,374; 2,841)	676 (155; 1,876)	5,058 (4,192; 5,941)
Aroma	13.1 (8.7; 22.9)	6.7 (3.9; 11.6)	4.0 (0.8; 15.3)	21.3 (14.7; 30.9)	3,119 (2,061; 5,458)	1,606 (918; 2,773)	960 (194; 3,651)	5,066 (3,499; 7,366)
Arque	20.7 (14.8; 28.2)	6.7 (3.6; 12.4)	2.4 (0.5; 9.5)	26.8 (19.8; 34.4)	3,389 (2,425; 4,615)	1,093 (587; 2,028)	398 (79; 1,552)	4,380 (3,233; 5,625)
Atahuallpa	13.1 (8.9; 23.1)	6.8 (3.6; 12.3)	3.7 (0.7; 13.1)	20.9 (15.4; 30.3)	487 (332; 859)	252 (135; 458)	137 (25; 487)	777 (574; 1,127)
Ayopaya	55.4 (44.8; 65.2)	12.2 (8.0; 18.3)	7.6 (2.1; 18.7)	60.3 (50.6; 69.5)	12,922 (10,443; 15,214)	2,835 (1,875; 4,271)	1,779 (498; 4,350)	14,061 (11,798; 16,212)
Bautista Saavedra	42.7 (34.9; 55.9)	11.5 (7.5; 17.4)	7.7 (1.8; 18.1)	48.8 (40.3; 60.8)	2,309 (1,889; 3,027)	624 (407; 942)	418 (97; 979)	2,639 (2,179; 3,288)
Belisario Boeto	37.9 (30.7; 45.6)	18.2 (11.8; 26.5)	7.2 (1.6; 19.4)	48.1 (39.2; 56.6)	1,011 (819; 1,218)	487 (315; 707)	193 (44; 518)	1,286 (1,047; 1,513)
Bernardino Bilbao	36.9 (28.3; 45.9)	9.6 (4.9; 16.9)	2.4 (0.3; 9.8)	42.0 (32.9; 51.6)	1,195 (918; 1,485)	309 (159; 546)	78 (11; 318)	1,360 (1,065; 1,671)
Burnet O'Connor	38.2 (32.0; 44.6)	21.2 (14.5; 29.7)	7.6 (1.5; 20.3)	50.2 (42.4; 57.5)	5,269 (4,412; 6,149)	2,923 (1,998; 4,090)	1,050 (211; 2,798)	6,914 (5,840; 7,928)
Capinota	36.9 (27.8; 47.7)	10.2 (4.4; 19.3)	1.2 (0.1; 7.0)	42.8 (31.9; 54.3)	5,373 (4,043; 6,950)	1,483 (647; 2,816)	170 (20; 1,020)	6,228 (4,646; 7,901)
Carangas	13.0 (8.8; 23.0)	7.0 (3.8; 12.3)	4.8 (1.0; 16.7)	22.0 (15.6; 32.3)	1,221 (825; 2,152)	656 (357; 1,157)	454 (91; 1,569)	2,064 (1,466; 3,024)
Carrasco	49.6 (41.4; 57.6)	26.4 (18.1; 36.4)	17.0 (5.4; 34.9)	61.4 (53.5; 69.7)	18,629 (15,537; 21,646)	9,907 (6,790; 13,657)	6,367 (2,043; 13,112)	23,066 (20,089; 26,191)
Cercado (Cochabamba)	18.9 (10.4; 29.6)	3.5 (0.8; 15.5)	0.1 (0.0; 1.2)	22.0 (12.0; 37.0)	9,445 (5,170; 14,784)	1,740 (408; 7,739)	58 (4; 579)	10,983 (5,991; 18,456)
Cercado (Oruro)	13.0 (8.8; 22.7)	6.6 (3.6; 11.3)	2.9 (0.5; 11.9)	20.5 (15.2; 29.2)	6,022 (4,068; 10,472)	3,065 (1,655; 5,230)	1,327 (249; 5,483)	9,453 (7,012; 13,483)
Cercado (Tarija)	14.8 (10.0; 23.4)	12.3 (7.0; 20.4)	10.2 (3.4; 18.5)	29.8 (21.1; 40.1)	4,217 (2,845; 6,681)	3,518 (1,998; 5,816)	2,909 (958; 5,270)	8,517 (6,019; 11,460)
Cercado (El Beni)	55.4 (45.7; 64.6)	35.7 (23.3; 50.5)	24.6 (7.5; 43.6)	70.7 (61.0; 79.2)	10,815 (8,912; 12,594)	6,958 (4,554; 9,858)	4,808 (1,460; 8,506)	13,790 (11,898; 15,444)
Chapare	48.5 (37.9; 59.5)	10.8 (6.9; 17.9)	4.5 (1.3; 10.0)	53.2 (42.3; 62.9)	73,219 (57,168; 89,884)	16,348 (10,484; 27,060)	6,756 (2,021; 15,029)	80,295 (63,844; 94,967)
Charcas	45.3 (37.8; 52.9)	11.3 (6.8; 17.5)	3.4 (0.7; 10.9)	50.2 (42.8; 58.0)	6,758 (5,638; 7,885)	1,688 (1,013; 2,610)	511 (109; 1,619)	7,488 (6,380; 8,653)
Chayanta	30.1 (24.9; 35.9)	8.3 (4.9; 14.2)	3.8 (0.8; 13.0)	36.3 (30.3; 43.6)	10,064 (8,328; 12,005)	2,786 (1,629; 4,751)	1,276 (284; 4,355)	12,136 (10,137; 14,572)
Chiquitos	46.0 (38.2; 54.2)	35 (22.7; 47.9)	24.4 (7.2; 47.0)	66.0 (55.1; 76.3)	21,253 (17,674; 25,037)	16,188 (10,489; 22,152)	11,288 (3,319; 21,720)	30,509 (25,482; 35,240)
Cordillera	29.5 (24.4; 34.6)	32.2 (22.1; 45.7)	22.9 (6.9; 43.7)	56.6 (46.2; 67.5)	10,494 (8,694; 12,327)	11,454 (7,886; 16,279)	8,168 (2,443; 15,574)	20,168 (16,455; 24,016)
Cornelio Saavedra	13.7 (9.4; 22.2)	9.2 (5.3; 15.2)	3.8 (0.8; 13.8)	23.3 (17.1; 32.0)	2,741 (1,891; 4,447)	1,840 (1,057; 3,039)	760 (155; 2,769)	4,670 (3,415; 6,403)
Daniel Campos	13.1 (8.8; 22.8)	7.0 (3.9; 12.3)	3.7 (0.8; 13.4)	21.3 (15.4; 30.1)	281 (189; 489)	150 (84; 264)	80 (16; 287)	456 (331; 645)
Eduardo Avaroa	13.1 (8.8; 23.3)	5.8 (3.2; 11.3)	3.5 (0.8; 12.9)	20.3 (14.2; 28.4)	2,090 (1,407; 3,715)	932 (516; 1,808)	563 (127; 2,058)	3,237 (2,256; 4,518)
Eliodoro Camacho	22.2 (16.1; 35.6)	6.1 (3.2; 12.0)	2.9 (0.6; 9.8)	28.0 (20.5; 40.5)	2,519 (1,831; 4,045)	695 (366; 1,364)	332 (66; 1,112)	3,173 (2,327; 4,592)
Esteban Arce	23.9 (17.4; 33.5)	9.6 (4.7; 16.6)	1.2 (0.1; 5.1)	30.6 (23.2; 40.2)	3,288 (2,393; 4,596)	1,324 (649; 2,284)	164 (18; 706)	4,200 (3,181; 5,524)
Eustaquio Méndez	14.1 (9.5; 23.9)	11.5 (6.9; 18.6)	8.9 (2.0; 18.5)	28.0 (19.7; 39.0)	2,233 (1,510; 3,792)	1,818 (1,091; 2,952)	1,417 (324; 2,943)	4,439 (3,128; 6,191)
Federico Román	55.1 (45.6; 64.0)	35.0 (22.7; 48.5)	38.2 (15.8; 56.9)	74.2 (64.4; 82.5)	6,896 (5,712; 8,012)	4,377 (2,840; 6,066)	4,778 (1,972; 7,121)	9,285 (8,055; 10,324)
Florida	20.4 (14.0; 30.9)	22.6 (14.1; 33.5)	16.7 (4.0; 39.3)	43.6 (30.9; 58.3)	4,238 (2,914; 6,404)	4,678 (2,931; 6,959)	3,458 (837; 8,150)	9,054 (6,405; 12,085)
Franz Tamayo	51.0 (42.1; 58.4)	21.2 (14.9; 29.7)	14.8 (4.9; 30.5)	60.3 (52.2; 68.1)	3,313 (2,738; 3,800)	1,378 (969; 1,930)	965 (321; 1,984)	3,922 (3,392; 4,429)
Germán Jordán	19.1 (8.8; 33.3)	9.6 (2.5; 22.4)	0.6 (0.0; 5.8)	26.6 (12.2; 43.9)	677 (312; 1,180)	341 (89; 794)	23 (1; 204)	943 (433; 1,557)
Gran Chaco	33.1 (28.0; 38.5)	31.2 (20.7; 43.5)	18.6 (5.2; 38.9)	57.5 (47.9; 68.6)	12,549 (10,624; 14,595)	11,850 (7,857; 16,507)	7,053 (1,974; 14,749)	21,822 (18,161; 26,007)
Gualberto Villarroel	13.1 (8.3; 23.2)	7.1 (3.6; 12.9)	5.7 (1.0; 21.3)	22.4 (14.3; 34.4)	947 (602; 1,686)	514 (260; 937)	414 (75; 1,546)	1,628 (1,035; 2,492)
Hernando Siles	55.2 (46.4; 64.5)	22.7 (15.9; 32.6)	6.0 (1.6; 16.4)	62.9 (54.7; 71.3)	4,443 (3,734; 5,190)	1,826 (1,280; 2,625)	482 (132; 1,317)	5,067 (4,401; 5,745)
Ichilo	54.4 (44.4; 64.8)	31 (20.1; 43.9)	24.1 (7.7; 48.7)	69.3 (58.5; 79.5)	28,867 (23,539; 34,362)	16,467 (10,639; 23,306)	12,788 (4,097; 25,829)	36,733 (31,045; 42,139)
Ignacio Warnes	56.5 (45.0; 67.6)	39.1 (24.5; 54.5)	19.5 (5.1; 45.5)	72.7 (60.5; 83.0)	22,228 (17,704; 26,608)	15,387 (9,642; 21,439)	7,683 (2,004; 17,912)	28,582 (23,817; 32,647)

Ingavi	14.9 (10.7; 22.7)	7.0 (4.2; 11.7)	1.9 (0.4; 6.5)	21.4 (16.4; 29.3)	4,608 (3,333; 7,051)	2,174 (1,303; 3,623)	593 (120; 2,004)	6,628 (5,086; 9,070)
Inquisivi	36.3 (30.3; 41.7)	9.6 (6.0; 14.8)	4.3 (1.0; 14.1)	41.5 (35.4; 48.6)	6,712 (5,600; 7,710)	1,778 (1,110; 2,732)	801 (180; 2,600)	7,678 (6,549; 8,984)
Jaime Zudáñez	14.6 (10.5; 23.0)	14.1 (9.2; 20.7)	4.3 (1.0; 13.5)	27.9 (21.3; 35.9)	1,306 (936; 2,058)	1,261 (819; 1,852)	386 (88; 1,206)	2,494 (1,903; 3,208)
José Ballivián	55.2 (45.9; 64.4)	34.3 (22.6; 48.0)	23.7 (10.4; 37.5)	70.3 (61.9; 77.8)	10,950 (9,111; 12,783)	6,809 (4,491; 9,513)	4,708 (2,058; 7,439)	13,944 (12,270; 15,437)
J. M. Avilés	14.5 (9.1; 25.8)	12.8 (7.2; 23.1)	8.8 (2.3; 20.0)	29.0 (19.4; 42.8)	2,048 (1,285; 3,635)	1,811 (1,022; 3,263)	1,247 (325; 2,823)	4,093 (2,743; 6,041)
J. M.Linares	13.2 (8.7; 22.9)	8.7 (5.2; 14.7)	4.5 (0.9; 14.3)	22.9 (16.2; 31.3)	1,483 (974; 2,570)	983 (583; 1,653)	501 (106; 1,605)	2,575 (1,823; 3,513)
J. M. de Velasco	55.2 (46.2; 64.2)	34.3 (22.7; 47.6)	24.0 (7.4; 43.4)	70.2 (61.3; 78.0)	11,240 (9,406; 13,069)	6,981 (4,627; 9,705)	4,895 (1,500; 8,833)	14,300 (12,485; 15,887)
J. A. de Padilla	31.9 (26.1; 39.0)	14.3 (8.9; 21.2)	4.5 (1.0; 14.5)	41.5 (34.5; 49.4)	2,071 (1,691; 2,533)	928 (580; 1,377)	293 (66; 939)	2,693 (2,239; 3,203)
Ladislao Cabrera	13.1 (8.9; 22.7)	7.2 (4.0; 12.8)	4.7 (1.1; 17.7)	22.0 (16.2; 32.1)	530 (360; 915)	291 (160; 516)	190 (42; 714)	889 (652; 1,295)
Larecaja	50.1 (41; 59.6)	20.2 (13.7; 28.9)	11.2 (2.9; 26.3)	58.8 (50.0; 67.1)	66,801 (54,729; 79,558)	26,941 (18,273; 38,599)	15,004 (3,861; 35,094)	78,370 (66,738; 89,516)
Litoral	13.2 (8.2; 23.2)	7.2 (4.0; 12.8)	5.0 (0.9; 17.8)	22.1 (14.7; 33.9)	124 (77; 219)	68 (38; 121)	47 (9; 168)	208 (139; 320)
Loayza	19.6 (14.9; 27.0)	9.0 (5.1; 15.4)	2.7 (0.5; 10.4)	27.2 (21.1; 35.2)	4,427 (3,364; 6,103)	2,039 (1,163; 3,473)	614 (120; 2,347)	6,157 (4,774; 7,973)
Los Andes	12.5 (8.0; 21.5)	5.5 (3.5; 9.8)	0.3 (0.1; 1.5)	17.3 (12.2; 26.3)	3,994 (2,570; 6,864)	1,759 (1,121; 3,137)	111 (22; 470)	5,539 (3,906; 8,427)
Luis Calvo	38.1 (32.1; 44.2)	28.0 (18.8; 39.3)	14.7 (4.2; 30.4)	57.6 (48.5; 67.2)	2,377 (2,002; 2,758)	1,744 (1,171; 2,453)	918 (263; 1,896)	3,590 (3,024; 4,195)
Madre de Dios	55.3 (45.9; 65.1)	36.0 (23.2; 49.4)	24.1 (7.1; 43.2)	70.6 (61.2; 79.1)	1,902 (1,577; 2,240)	1,238 (799; 1,700)	829 (246; 1,486)	2,428 (2,104; 2,718)
Mamoré	55.4 (45.4; 64.6)	36.0 (23.6; 49.7)	27.0 (8.9; 46.8)	71.4 (62.0; 79.9)	2,478 (2,031; 2,888)	1,610 (1,053; 2,224)	1,207 (396; 2,093)	3,192 (2,770; 3,573)
Manco Kapac	55.9 (36.7; 70.9)	6.2 (2.6; 14.9)	1.2 (0.1; 9.8)	58.0 (38.5; 73.3)	3,945 (2,587; 5,004)	440 (181; 1,049)	84 (7; 694)	4,092 (2,718; 5,172)
M. M. Caballero	35.1 (29.5; 41.2)	19.3 (13.0; 27.1)	9.6 (2.6; 21.8)	47.3 (40.2; 54.5)	2,831 (2,381; 3,324)	1,557 (1,048; 2,188)	774 (209; 1,762)	3,821 (3,244; 4,403)
Manuripi	55.4 (45.6; 64.5)	35.1 (23.1; 49.1)	25.9 (8.3; 45.1)	71.2 (61.7; 79.7)	1,607 (1,323; 1,869)	1,018 (671; 1,423)	751 (241; 1,308)	2,066 (1,789; 2,310)
Marbán	55.3 (45.5; 64.3)	35.1 (23; 49.4)	24.6 (7.4; 44.1)	70.3 (61.4; 79.1)	8,160 (6,717; 9,478)	5,182 (3,385; 7,291)	3,629 (1,091; 6,509)	10,368 (9,052; 11,666)
Mizque	35.7 (28.6; 41.8)	12.0 (7.6; 18.4)	2.9 (0.7; 10.3)	42.5 (35.0; 49.6)	3,709 (2,978; 4,345)	1,244 (787; 1,914)	299 (69; 1,069)	4,419 (3,637; 5,160)
Modesto Omiste	13.1 (8.3; 22.7)	8.1 (4.4; 13.7)	4.5 (0.8; 14.2)	22.5 (15.6; 31.5)	960 (608; 1,660)	592 (323; 1,000)	330 (60; 1,037)	1,645 (1,140; 2,302)
Moxos	55.5 (46.1; 64.9)	34.9 (22.5; 48.9)	23.7 (7.1; 43.2)	70.1 (60.3; 79.0)	7,380 (6,136; 8,632)	4,642 (2,990; 6,510)	3,158 (946; 5,743)	9,331 (8,020; 10,508)
Muñecas	42.3 (34.2; 51.7)	10.5 (6.3; 16.8)	4.2 (0.8; 12.4)	47.2 (38.8; 56.8)	2,770 (2,240; 3,383)	689 (413; 1,101)	276 (52; 815)	3,091 (2,543; 3,717)
Narciso Campero	16.0 (11.8; 24.4)	16.4 (11.2; 23.7)	4.3 (1.0; 13.1)	30.6 (24.5; 38.7)	1,985 (1,470; 3,030)	2,042 (1,397; 2,952)	533 (125; 1,627)	3,798 (3,043; 4,811)
Nor Chichas	13.0 (8.7; 22.4)	9.0 (5.2; 14.2)	5.3 (1.1; 17.0)	23.6 (17.3; 32.5)	961 (645; 1,653)	665 (385; 1,044)	389 (85; 1,257)	1,741 (1,276; 2,398)
Nor Cinti	14.9 (11.0; 23.3)	10.9 (6.8; 16.2)	5.3 (1.1; 15.9)	26.1 (20.0; 33.8)	2,252 (1,668; 3,532)	1,648 (1,033; 2,452)	811 (164; 2,411)	3,958 (3,033; 5,124)
Nor Lípez	13.1 (9.0; 22.7)	6.7 (3.8; 12.1)	3.5 (0.7; 12.3)	20.7 (15.3; 29.4)	718 (496; 1,248)	366 (206; 665)	190 (41; 674)	1,139 (839; 1,617)
Nor Yungas	53.0 (42.1; 64.5)	19.9 (13.4; 28.4)	9.5 (2.7; 23.5)	61.8 (51.2; 72.5)	21,583 (17,155; 26,242)	8,095 (5,435; 11,574)	3,885 (1,101; 9,575)	25,148 (20,862; 29,507)
Ñuflo de Chávez	54.8 (45.4; 63.6)	34.2 (22.4; 48.6)	24.8 (7.7; 45.0)	70.2 (60.7; 78.6)	25,700 (21,275; 29,827)	16,019 (10,519; 22,788)	11,632 (3,601; 21,122)	32,915 (28,483; 36,843)
Obispo Santistevan	55.2 (46.2; 64.7)	35.2 (23.0; 49.2)	24.3 (7.5; 46.7)	70.6 (61.1; 80.2)	18,466 (15,468; 21,634)	11,783 (7,692; 16,467)	8,119 (2,521; 15,621)	23,614 (20,440; 26,832)
Omasuyos	15.2 (10.5; 22.5)	8.1 (5.0; 12.5)	0.5 (0.1; 2.7)	22.0 (16.1; 28.5)	3,774 (2,599; 5,562)	2,007 (1,228; 3,099)	135 (13; 657)	5,451 (3,992; 7,070)
Oropeza	33.5 (26.8; 40.4)	11.9 (7.3; 18.4)	3.1 (0.6; 12.3)	41.4 (34.2; 48.7)	17,185 (13,745; 20,716)	6,110 (3,755; 9,413)	1,574 (315; 6,331)	21,239 (17,542; 25,002)
Pacajes	13.4 (9.1; 22.3)	6.1 (3.4; 10.7)	5.0 (1.0; 16.9)	22.0 (15.7; 31.5)	2,276 (1,540; 3,796)	1,033 (576; 1,821)	855 (174; 2,880)	3,733 (2,670; 5,350)
Pantaleón Dalence	13.0 (7.5; 24.0)	5.8 (2.8; 12.3)	0.6 (0.1; 5.1)	18.3 (11.4; 29.2)	780 (451; 1,441)	351 (169; 740)	36 (5; 304)	1,097 (684; 1,753)
P. D. Murillo	23.3 (18.8; 29.5)	9.1 (6.1; 13.1)	2.8 (0.6; 7.8)	29.0 (24.1; 35.2)	54,432 (44,011; 68,903)	21,185 (14,271; 30,607)	6,454 (1,495; 18,242)	67,673 (56,221; 82,183)
Poopó	13.0 (8.6; 23.1)	6.4 (3.3; 12.5)	3.1 (0.5; 12.8)	20.3 (14.0; 29.7)	759 (503; 1,347)	376 (193; 727)	180 (32; 747)	1,184 (816; 1,733)
Punata	13.5 (6.2; 26.0)	9.9 (4.0; 21.4)	0.9 (0.1; 7.3)	22.1 (11.2; 40.2)	761 (350; 1,465)	558 (223; 1,204)	53 (4; 411)	1,245 (632; 2,264)
Quillacollo	46.8 (36.2; 56.5)	7.0 (2.7; 13.9)	0.9 (0.1; 4.7)	50.1 (39.2; 60.3)	26,104 (20,213; 31,482)	3,916 (1,517; 7,749)	495 (67; 2,641)	27,916 (21,835; 33,602)
Rafael Bustillo	13.1 (8.4; 22.8)	6.5 (3.5; 11.4)	3.6 (0.7; 12.0)	20.6 (14.2; 29.9)	1,881 (1,208; 3,290)	935 (499; 1,640)	516 (107; 1,731)	2,974 (2,050; 4,314)
Sajama	13.1 (8.9; 23.2)	6.2 (3.3; 11.1)	3.5 (0.6; 14.6)	20.5 (14.8; 30.1)	472 (319; 833)	222 (120; 400)	127 (21; 526)	736 (532; 1,084)
Sara	56.4 (44.9; 67.0)	38.6 (26.5; 53.6)	20.3 (5.7; 47.4)	72.8 (61.7; 83.4)	33,743 (26,889; 40,095)	23,061 (15,858; 32,034)	12,172 (3,392; 28,381)	43,552 (36,912; 49,864)
Saucarí	13.1 (8.3; 24.5)	7.4 (3.8; 13.4)	4.9 (1.0; 17.9)	22.4 (15.0; 34.1)	786 (501; 1,470)	442 (230; 804)	296 (59; 1,077)	1,344 (903; 2,049)
Sud Chichas	12.9 (8.9; 22.5)	7.7 (4.3; 13.1)	5.0 (1.0; 14.7)	22.4 (16.0; 31.2)	1,302 (896; 2,266)	772 (431; 1,319)	508 (97; 1,478)	2,253 (1,616; 3,144)
Sud Cinti	21.8 (17.6; 28.0)	15.1 (10.3; 21.6)	6.7 (1.3; 17.1)	34.1 (27.4; 41.5)	1,363 (1,105; 1,753)	948 (645; 1,352)	419 (84; 1,072)	2,135 (1,719; 2,601)

Sud Lípez	13.0 (8.9; 22.5)	4.7 (2.4; 9.6)	0.7 (0.2; 4.8)	17.5 (12.9; 26.4)	288 (197; 499)	105 (53; 214)	16 (3; 108)	389 (288; 587)
Sud Yungas	36.9 (30.5; 44.4)	15.8 (10.8; 23.0)	6.3 (2.2; 13.1)	44.7 (37.8; 52.5)	16,409 (13,577; 19,740)	7,021 (4,794; 10,247)	2,784 (961; 5,805)	19,878 (16,818; 23,327)
Tapacarí	40.7 (31.8; 49.4)	7.2 (3.4; 13.1)	2.4 (0.4; 10.8)	44.7 (35.4; 53.9)	8,080 (6,311; 9,807)	1,421 (667; 2,602)	479 (85; 2,137)	8,873 (7,028; 10,703)
Tomás Frías	13.1 (8.7; 23.0)	6.3 (3.3; 12.1)	3.8 (0.9; 14.0)	20.8 (14.3; 30.3)	5,484 (3,668; 9,637)	2,665 (1,381; 5,059)	1,587 (364; 5,874)	8,727 (5,998; 12,716)
Tomina	36.9 (30.7; 43.0)	16.7 (11.0; 24.3)	6.3 (1.6; 16.8)	46.6 (39.9; 53.8)	3,452 (2,871; 4,019)	1,562 (1,029; 2,271)	588 (149; 1,571)	4,358 (3,730; 5,034)
Vaca Díez	55.2 (46.5; 63.9)	35.8 (23.7; 49.5)	45.1 (19.2; 64.3)	76.0 (65.5; 84.9)	22,559 (18,993; 26,125)	14,651 (9,691; 20,253)	18,415 (7,848; 26,297)	31,067 (26,787; 34,705)
Vallegrande	40.9 (34.0; 47.9)	19.4 (13.0; 27.4)	7.0 (1.5; 19.5)	51.0 (44.0; 58.1)	2,674 (2,218; 3,126)	1,264 (846; 1,789)	455 (101; 1,273)	3,334 (2,871; 3,797)
Yacuma	55.3 (46.0; 63.9)	35.8 (23.2; 49.7)	33.9 (13.1; 51.1)	73.0 (63.9; 81.2)	4,597 (3,826; 5,313)	2,978 (1,927; 4,129)	2,818 (1,091; 4,248)	6,067 (5,311; 6,748)
Yamparáez	16.1 (9.4; 27.8)	10.9 (6.4; 17.9)	3.3 (0.5; 13.0)	26.3 (17.7; 37.4)	1,841 (1,072; 3,180)	1,243 (730; 2,051)	379 (59; 1,486)	3,008 (2,026; 4,274)
**Country**								
Bolivia	38 (32.4; 43.4)	19.3 (14.2; 25.9)	11.4 (3.6; 23.7)	48.4 (43.1; 54.4)	[illegible]1,453 (801,990; 1,073,112)	[illegible]478,856 (352,584; 642,099)	281,448 (89,989; 586,020)	[illegible]182 (1,067,216; 1,347,507)

* calculated based on the 5-14 years old population for 2010

** calculated under the assumption of independenc of *A. lumbricoides* , *T.trichiura* and hookworm infections
